# Supplementary material for: Are carbon emissions trading and green financial instruments synergistic? -Comprehensive quantitative research based on content analysis
Source: PLoS One. 2024 Mar 7;19(3):e0298601. doi: 10.1371/journal.pone.0298601 (PMC10919601; doi:10.1371/journal.pone.0298601)
Supplement: S1 Data — (ZIP) [file pone.0298601.s001.zip › Data/地级市Z_F工作报告.zip!/▒▒╛⌐/└┤╫╘╣1⁄2╓┌║┼í╛┬φ┐╦╩2╛▌═°í┐_2008.docx]

——2008年1月20日在北京市第十三届人民代表大会第一次会议上

北京市代市长 郭金龙

各位代表：

　　现在，我代表北京市人民政府向大会作政府工作报告，请予审议，并请市政协各位委员提出意见。

　　一、五年工作的回顾

　　北京市第十二届人民代表大会第一次会议以来，全市人民在中共北京市委的领导下，坚持以邓小平理论和“三个代表”重要思想为指导，深入贯彻落实科学发展观，紧紧围绕实现“新北京、新奥运”战略构想，抓住机遇，求真务实，锐意进取，首都改革开放和现代化建设取得新的重大成就。“十五”计划胜利完成，“十一五”规划进展顺利。

　　——首都经济迈上新台阶。初步统计，地区生产总值由2002年的4330.4亿元增长到2007年的9006.2亿元，年均增长12%以上，人均地区生产总值突破7000美元。地方财政收入由534亿元增长到1492.6亿元，增长1.8倍。万元地区生产总值能耗、水耗以及化学需氧量、二氧化硫排放量逐年下降，节能减排走在全国前列。

　　——改革开放更加深入。农村、国有企业和投资、金融、财税、科技等领域改革取得重大进展，非公有制经济进一步发展。累计实际利用外资183.6亿美元，是前五年的1.8倍；2007年地区进出口总额1929.5亿美元，是2002年的3.7倍。

　　——城乡面貌日新月异。交通建设跨越式发展，公交出行比例达到34.5%，比2002年提高6.5个百分点。市区空气质量二级以上天数达到67.4%，比2002年提高11.8个百分点；城八区和郊区污水处理率分别达到92%和47%，生活垃圾无害化处理率分别达到99%和76%；全市林木绿化率达到51.6%。

　　——奥运筹备顺利推进。26个比赛场馆、44个独立训练场馆竣工。“好运北京”系列赛事成功举办，各项保障工作有序展开。城市环境质量和市民文明素质不断提高。

　　——社会建设开创新局面。教育事业全面进步，科技创新能力明显增强。覆盖城乡居民的社会保障制度框架初步形成。首都公共卫生、社区卫生服务体系基本建立。文化更加繁荣，体育蓬勃发展。

　　——人民生活显著改善。城镇居民人均可支配收入、农民人均纯收入分别达到21989元和9559元，年均实际增长11.5%和9.1%。五年全市增加就业263万人，登记失业率始终控制在2.3%以内。城镇居民人均住房使用面积超过20平方米。

　　五年来，本届政府以科学发展观为指导，重点做了以下几方面工作：

　　(一)着力转变经济发展方式，促进首都经济又好又快发展。认真落实中央宏观调控政策措施，投资结构不断优化，城乡居民消费能力逐步增强，全社会固定资产投资、社会消费品零售额分别增长了2.2倍和1.9倍，投资、消费拉动趋于协调。严格清理固定资产投资项目，全面清理整顿各类开发区。完成第一次经济普查和第二次农业普查，摸清了全市二、三产业和“三农”的基本情况。确立高端产业发展战略，加快发展生产性服务业、文化创意产业、高技术产业和现代制造业，重点推进六大高端产业功能区建设。服务型经济主导地位进一步巩固，第三产业比重达到71%以上。制定实施循环经济发展规划，强化节能、节水、节地、节材各项措施，推动大型公建节能和非节能建筑改造，下大力气调整退出高耗能、高排放企业，稳步推进首钢等企业搬迁，坚决关闭小煤矿、非煤矿山和砂石生产企业，重点支持石化、建材、电力等行业开展清洁生产，工业节能降耗成效明显。首都经济发展呈现速度较快、结构趋优、消耗降低、效益提高的良好态势。

　　(二)统筹城乡发展，扎实推进社会主义新农村建设。2007年市政府对“三农”投入130.6亿元，是2002年的5.2倍。建立了“部门联动、政策集成、资金聚焦、资源整合”的新农村建设工作机制，各部门工作重点向农村延伸。实施“221行动计划”，加强农业服务体系建设，大力发展农民专业合作组织，建立农业政策性保险制度，率先实施农业生态服务补贴，促进都市型现代农业发展。加快村庄规划编制，加强基础设施建设，实施“农村亮起来、农民暖起来、农业资源循环起来”工程，所有行政村铺通了柏油路，广播电视实现“村村通”，3万多农户搬出泥石流易发区和采空区，农民安全饮水达标率由36%提高到79%，农村生产生活条件有了很大改善。全面落实国家各项惠农政策。完成土地承包经营权确权工作，规范建设征地补偿办法，完善被征地农民的社会保障政策，提高生态林建设占地补偿和养护补助标准。建立山区生态林补偿机制和农村管水员、乡村公路养护员制度，市级财政累计投入5.7亿元，帮助6万多农民在公益性岗位稳定就业。不断加大新型农民培养力度，实行村级经费补贴制度，全面发展农村社会事业。这五年，是制定实施“三农”政策最多、力度最大、农民得实惠最多的五年。

　　(三)加强城乡规划建设管理，大力提升城市承载能力和服务水平。在首都规划委员会的指导下，完成城市总体规划修编，确立了“国家首都、国际城市、文化名城、宜居城市”的功能定位和“两轴—两带—多中心”的城市空间结构。制定区县功能定位及评价指标体系，统筹协调发展的格局逐步形成。高度重视古都风貌保护，完成一批重要文物建筑的修复，探索实施政府主导的旧城保护修缮和有机更新模式。完善城乡路网结构，实施市区交通疏堵工程，路网通行能力有效提高。五年新增城市道路669公里、高速公路162公里，改造提级公路8770公里，建成全国首条大容量快速公交线路。轨道交通新增运营里程66.6公里，总里程达到142公里，网络化运营格局初步形成。坚持公共交通公益性定位，实行低票价政策，优化公交线路，全面推行市政交通一卡通，公交整体发展水平显著提升。国家大剧院、首都博物馆等一批标志性建筑先后建成。基本完成南水北调北京段主体工程。坚持不懈地治理大气污染，实施控制大气污染第九至十三阶段措施，运用综合手段改善市区空气质量。建成12座污水处理厂和一批农村污水处理设施，完成城市骨干河道治理，水环境得到有效保护。城区基本实现生活垃圾无害化处理，农村垃圾“村收集、镇运输、区处理”新机制全面推行。危险废物无害化处置设施不断完善，建成放射性废物库。实施废弃矿山生态修复，营造防风固沙林7万多公顷，三道绿色生态屏障基本建成，城乡绿化美化取得显著成效。发挥市民主体作用，创新管理方式，建设网格化管理平台，提升信息化水平，城市服务管理能力大幅提高，在烟花爆竹“禁改限”、中非合作论坛服务保障等工作中得到体现。

　　(四)高标准做好各项筹备工作，为成功举办2008年奥运会、残奥会奠定坚实基础。坚决贯彻落实中央指示精神，全面实践“绿色奥运、科技奥运、人文奥运”三大理念，不断提高各项筹备工作水平。优化奥运场馆规划设计，确立“安全、质量、工期、功能、成本”五统一原则，奥运工程建设顺利进行。突出环保、节能要求，广泛采用新技术、新工艺、新设备，在钢结构、膜结构等领域取得多项自主创新成果，谱写了中国建筑史上的新篇章。城市交通、公共安全、水电气热供应、无障碍设施、住宿餐饮、商业服务、社会秩序等各项保障工作有序推进，场馆运行、外围保障和城市运行无缝衔接的体制机制基本建立。全面开展城乡环境综合整治，市容市貌大为改观。广泛开展“迎奥运、讲文明、树新风”活动，实施“五大文明行动”，创建公共文明示范地区，推进窗口行业单位奥运培训，举办全民健身与奥运同行、市民讲外语等群众文体活动，全社会参与奥运、奉献奥运、共享奥运的氛围日渐浓厚。

　　(五)注重提高自主创新能力，加快建设创新型城市。深化科技体制改革，实施科技主题计划，组织了210个重大科技项目，科技支撑首都发展的能力明显增强。设立高成长企业自主创新专项资金，支持搭建科技条件平台，促进企业技术中心建设，企业创新主体地位得到强化。专利申请量稳步增长，发明专利比重超过50%。加快技术转移和交易体系建设，2007年全市技术合同成交额达882.6亿元。推动自主创新与实施品牌战略相结合，涌现出了一批中国名牌产品。优化高等教育和职业教育专业结构，鼓励开展多种形式的产学研合作，支持大学科技园和孵化器建设。完善鼓励留学人员归国创业的政策措施，形成吸引人才的有效机制，首都创新型人才更加聚集。出台了建设创新型城市的实施意见及配套措施，自主创新环境不断优化。

　　贯彻落实国务院关于做强中关村科技园区的重要决策，“五年上台阶”各项任务顺利完成。开展非上市公司代办股份转让等试点，以信用为基础、多渠道融资的投融资体系初步建立。

　　整合创新资源，启动百家创新型企业试点，推动建立“闪联”等23个产业技术联盟，企业协同创新能力不断提升，涌现出一批新技术、新产品和新标准。加强基础设施和公共服务设施建设，国家级产业基地及专业园区建设进展顺利，创新集群正在形成。

　　(六)深入推进改革开放，不断优化发展环境。对市与区县财政管理体制进行了调整，预算管理、国库管理等制度得到完善，公共支出占财政支出比重由2002年的81.8%提高到2007年的90.5%。加快投融资体制改革，试行政府投资项目储备、公益性项目代建、重大项目公示等制度。强化国有资产监督管理，完成国有资产清产核资，探索建立国有资本经营预算制度，推进国有企业“调改剥退”，国有经济布局结构逐步优化。通过实施重组、引进战略投资者等改革举措，有效化解金融风险，市属金融企业实力显著增强。贯彻落实《行政许可法》，精简行政审批、收费和企业年检事项，规范审批行为和程序。推广“一站式”办公和全程办事代理制，完善行政投诉工作机制。成立市政府对外联络办公室，积极做好为中央在京企事业单位的服务工作。整顿和规范市场秩序，出台了商业企业促销行为规范和供货商、零售商交易行为规范，认真开展治理商业贿赂专项工作，严厉打击侵权盗版和商业欺诈行为，信用体系建设进一步推进。清理限制性规定，搭建融资服务平台，鼓励支持非公经济发展。开展专业化、区域针对性强的投资促进活动，建立外商投资项目审批绿色通道。努力推进“大通关”，促进加工贸易转型升级和自主知识产权产品、自主品牌产品出口，大力发展服务贸易。2007年接待海外旅游者435.5万人次，旅游外汇收入达到45.8亿美元。拓展对外交流，国际友好城市达到41个，科博会、文博会、京港洽谈会、京台科技论坛、诺贝尔奖获得者北京论坛等大型活动越办越好。对口支援、京津冀区域协作迈出了新的步伐。

　　(七)切实解决民生问题，加快发展社会事业。完善各项促进就业政策，着力帮扶城乡就业困难群体，基本消除城乡零就业家庭。深入推行劳动合同和集体合同制度，较好地解决了农民工工资拖欠问题。实施农村社会养老保险、新型农村合作医疗和农村最低生活保障制度，农村社会保障体系逐步建立。全市城镇基本养老、基本医疗、失业保险覆盖率达到92%以上，建立生育保险、城镇居民“一老一小”大病医疗保险制度，农民工参加工伤和大病医疗保险人数分别达到190万和166万。稳步提高城乡居民社会保障相关待遇标准，城乡社会救助体系基本建立，社会福利事业、慈善事业加快发展。加大住房保障力度，累计建成经济适用住房1400万平方米，通过实物配租和发放租房补贴，解决了2.6万户最低收入家庭的住房困难。

　　建立和完善义务教育经费保障机制，提高公用经费定额标准，落实“两免一补”政策并扩大覆盖范围，完善家庭经济困难学生资助政策体系。采取多种有效措施，保障流动人口子女受教育权利。加快中小学规范化、标准化建设，开展城乡对口支援，改善农村学校、薄弱学校办学条件。高等教育率先进入普及化阶段，学习型城市建设稳步推进。加强文化活动基础设施建设，广泛举办群众喜闻乐见的文化活动，基本建成了覆盖城乡的公共文化服务体系。重点培育北京国际音乐节、新年音乐会、国际戏剧舞蹈音乐演出季等文化品牌。积极开展非物质文化遗产保护工作。大力推动文化体制改革，加快新闻出版、广播影视、文学艺术、哲学社会科学等各项事业发展。

　　在中央的正确领导下，依靠首都和部队医务人员舍生忘死的工作、广大市民的共同奋斗和全国人民的大力支持，取得抗击非典的重大胜利。加强艾滋病等重大疾病预防与控制，有效防控高致病性禽流感疫情。积极推广中医药服务。加快城乡社区卫生服务体系建设，建成167个标准化社区卫生服务中心和2137个社区卫生服务站，完成规划设置的70%。实行社区卫生服务机构收支两条线管理，对312个品种常用药采取集中采购、统一配送和零差率销售，社区药品平均价格下降30%。

　　(八)大力抓好安全工作，坚决维护首都安全稳定。牢固树立安全责任重于泰山的意识，建立和完善社会公共安全、生产安全、食品药品安全和公共卫生安全监控责任体系，积极探索建立安全工作长效机制。组建市区两级应急管理机构，制定突发公共事件各级各类应急预案，加强各类设施和专业化应急队伍建设，普及公共安全知识，基本建成应急管理体系，及时妥善处置了各类突发事件。建立健全安全隐患排查整改工作机制，累计消除市级安全隐患1587项。加强安全生产法制和制度建设，出台了人员密集场所安全管理的政府规章和多领域安全管理规范，全面落实行业监管、综合监管和属地监管责任及生产经营单位主体责任，加强对煤矿、非煤矿山、危险物品、人员密集场所的监管，安全生产形势总体稳定。建立全市统一的食品安全监控和追溯体系，有效控制了突发食品安全事件。加强药品全过程监管，建立生产经营单位退出机制，坚决治理虚假广告，保证了群众用药安全。加强矛盾纠纷排查化解，做好人民来信来访和行政复议工作，注重人民调解、行政调解和司法调解相衔接。不断完善社会治安防控体系，深入开展基层安全创建和平安建设活动，全面推进科技创安工程，严厉打击各类违法犯罪活动，积极开展社区矫正试点工作。坚决开展同“法轮功”等邪教组织的斗争，严密防范国内外敌对势力的破坏活动，首都社会秩序保持平稳。

　　(九)不断加强政府自身建设，努力提高服务管理水平。完成政府机构改革工作，调整了交通、水务、园林绿化等7个部门的管理体制，行政执法体系不断完善，乡镇和街道管理体制改革稳步推进，事业单位行政许可职能逐步剥离，支持行业协会等社会中介组织加快发展。深入贯彻国务院全面推进依法行政实施纲要，切实加强政府法制建设，五年共提请市人大常委会审议通过地方性法规41项，制定、修改和废止政府规章260项。认真贯彻监督法，全面执行人大及其常委会的决议，坚持重大事项报告制度，做好向人大常委会专项工作报告。主动加强与人民政协的联系，积极听取各民主党派、工商联、无党派人士和人民团体的意见。坚持与人大代表、政协委员的联系制度，五年共办复全国和市人大代表议案、建议9257件，政协委员建议案、提案6713件。完善重大决策的规则和程序，不断提高决策的科学化、民主化水平。加快推进政务公开和电子政务建设。压缩政府部门经常性项目支出资金，全面规范公务员收入。扎实开展先进性教育活动，加强领导干部作风建设，切实树立“八个方面”的良好风气。认真落实党风廉政建设责任制，坚决查处违纪违法案件，积极配合中纪委严肃查处了刘志华严重违纪问题。严格实施公务员法，提出“职责清、情况明、数字准、作风正、效率高”的要求，狠抓基层基础工作，努力为基层、企业和群众提供优质服务。

　　五年来，社会主义民主法制和精神文明建设成效显著。基层民主进一步扩大，村民自治、社区自治和村务、厂务公开逐步推行。普法宣传教育活动更加深入，市民的法制观念不断增强。广泛开展社会主义荣辱观教育，不断提升精神文明创建活动水平，未成年人思想道德建设和大学生思想政治工作进一步加强。民族、宗教、侨务和对台工作取得新成绩。人口和计划生育工作扎实推进。妇女儿童、老龄、残疾人事业健康发展。国防教育和国防后备力量建设得到加强，双拥共建工作深入扎实，同呼吸、共命运、心连心的军政军民关系进一步巩固。

　　过去的五年，是全市人民按照科学发展观的要求，抓住奥运机遇，创新体制、调整结构、优化环境、全面发展的五年；是扎实推进社会主义和谐社会建设，着力解决民生问题，广大人民群众普遍受益的五年；是万众一心，奥运筹备工作按计划推进，成果显著的五年；是城乡面貌发生巨大变化，城市服务功能日益增强，现代化水平和文明程度不断提高的五年。这些成绩的取得，是党中央、国务院正确指引，市委坚强领导的结果，是全市人民团结奋斗、不懈努力的结果。在这里，我代表市政府，向全市各族人民，向人大代表、政协委员，向各民主党派、各人民团体、社会各界人士，向中央各部门、兄弟省市区和驻京部队，向来自全国各地的劳动者，向所有关心支持首都建设的香港与澳门特别行政区同胞、台湾同胞、海外侨胞和国际友人，表示崇高的敬意和衷心的感谢！

　　各位代表，经过五年的实践，我们对推进首都改革开放和现代化建设的认识不断加深：

　　一是必须坚决与党中央保持高度一致，全面贯彻落实中央的各项大政方针和对北京工作的一系列指示精神，讲政治、顾大局，在任何时候、任何情况下，都要自觉维护中央权威，确保中央政令在北京畅通。

　　二是必须坚持解放思想、实事求是、与时俱进，从首都城市性质和功能出发，不断深化对首都经济规律、社会规律、自然规律的认识和把握，集中各方面的力量和智慧，全面履行好“四个服务”职能。

　　三是必须深入贯彻落实科学发展观，坚持首都经济的发展方向，不断丰富首都经济内涵，紧紧抓住奥运机遇，提高自主创新能力，切实转变经济发展方式，加快产业结构调整，着力化解人口资源环境矛盾，促进首都经济又好又快发展。

　　四是必须坚持立党为公、执政为民，把实现好、维护好、发展好人民群众的根本利益作为一切工作的出发点和落脚点，更加注重社会建设，着力保障和改善民生，使广大人民群众共享改革发展成果，努力构建社会主义和谐社会首善之区。

　　五是必须坚持社会主义市场经济发展方向，坚定不移地加快改革开放，充分发挥市场在资源配置中的基础作用，着力优化发展环境，为实现科学发展提供体制机制保障，使首都的资源优势持续不断地转化为发展优势。

　　六是必须加快服务型政府建设，努力探索城市政府履行职能的具体方式，统筹做好“规划、发展、建设、运行、服务、管理”六个方面工作，不断提升城市服务管理水平。

　　在总结成绩和经验的同时，我们也清醒地看到，目前首都经济社会发展中还存在一些突出的困难和问题，主要表现在：人口资源环境矛盾尚未得到缓解，治理环境污染、交通拥堵的任务仍很艰巨；公共服务能力亟需增强，城市管理水平亟待提高；经济发展方式需要进一步转变，城乡、区域差距较大，经济社会发展不平衡；住房市场结构不合理，价格上涨过快，违法违规用地时有发生；劳动就业、社会保障、收入分配、教育卫生、安全生产等方面关系群众切身利益的问题需要加快解决；政府职能转变还不到位，服务意识、工作作风、工作效率等方面还有明显差距，极少数干部以权谋私、贪污受贿等腐败现象依然存在。对于上述问题，必须高度重视，继续认真加以解决。

　　二、今后五年奋斗目标和2008年工作任务的建议

　　党的十七大高举中国特色社会主义伟大旗帜，对继续推进改革开放和社会主义现代化建设、实现全面建设小康社会的宏伟目标作出了全面部署，为做好首都工作指明了道路和方向。今后五年，北京将处于城市化、信息化、市场化、国际化的加速期。机遇前所未有，挑战也前所未有，机遇大于挑战。我们要在成功举办一届有特色、高水平奥运会的基础上，全面实现“十一五”规划，建设繁荣、文明、和谐、宜居的首善之区。根据市第十次党代会的部署，今后五年全市经济社会发展的奋斗目标和主要任务是：

　　——努力实现首都经济又好又快发展。完善现代市场体系，更好地发挥市场在资源配置中的基础性作用，建立健全充满活力、富有效率、更加开放的体制机制。切实转变发展方式，进一步优化产业结构，第三产业比重达到74%以上，提升现代制造业整体素质和竞争力，大力发展都市型现代农业，资源能源节约走在全国前列。显著增强自主创新能力，基本建成创新型城市。加快城市南部、西部地区建设，加强生态涵养发展区建设，全面推进新农村和新城建设，形成城乡经济社会发展一体化新格局。地区生产总值年均增长9%左右，人均地区生产总值达到10000美元以上。

　　——不断提高城市文明程度。大力发展社会主义先进文化，覆盖城乡的公共文化服务体系更加完善，妥善保护古都风貌，文化中心的功能不断加强。全面推进义务教育均衡发展，不断提高教育发展质量，基本实现教育现代化，建设学习型城市。深入落实依法治国基本方略，进一步增强全社会法制观念，推进法治政府建设，健全基层民主制度。不断完善精神文明建设的长效机制，使城市文明程度和市民文明素质明显提高。

　　——全面推进和谐社会首善之区建设。实现劳动者比较充分的就业，基本建立覆盖城乡的社会保障体系。实现基本公共服务均等化。收入分配秩序进一步规范，城乡居民家庭财产普遍增加。人民群众利益表达渠道更加畅通，基层化解社会矛盾的体制机制更加健全，首都社会更加安定有序。

　　——加快建设现代宜居城市。积极探索有效调控人口规模的政策、途径和方法，努力提高人口素质。大力推进生态文明建设，努力形成符合可持续发展要求的产业结构、增长方式、消费模式。增强基础设施承载能力，建设现代化的综合交通、能源供应、水资源供给体系。加强生态保护与环境治理，逐步建立起现代化的垃圾、污水处理体系，城市环境质量基本达到国家标准。

　　2008年是贯彻落实党的十七大精神的第一年，是北京奥运之年，我们将迎来改革开放30周年。做好2008年政府工作，责任十分重大。现在距离奥运会开幕只有201天，各项筹备和服务保障工作到了最关键的时候，决胜在即，时不我待，务必夺取奥运攻坚战的全面胜利。我们一定要切实增强紧迫感、责任感、使命感，充分发挥各方面的积极性、主动性、创造性，确保一届有特色、高水平的奥运会圆满成功！我们一定要保持经济社会的良好发展势头，不断巩固文明建设成果，努力营造经济发展、社会祥和的良好局面，让“新北京、新奥运”战略构想胜利实现！

　　2008年政府工作总的要求是：全面贯彻落实党的十七大精神，高举中国特色社会主义伟大旗帜，以邓小平理论和“三个代表”重要思想为指导，深入贯彻落实科学发展观，全力办好有特色、高水平的奥运会、残奥会，深化改革开放，推进自主创新，着力优化经济结构和提高经济增长质量，切实加强节能减排和生态环境保护，不断提升城市现代化建设与服务管理水平，更加重视改善民生和促进社会和谐，在新的起点上推动首都经济社会又好又快发展。

　　全市经济社会发展主要目标是：万元地区生产总值能耗、水耗分别下降5%和4%，化学需氧量和二氧化硫排放量分别下降4%和10%，市区空气质量二级以上天数力争达到70%；城镇登记失业率控制在2.3%以内；居民消费价格指数低于全国平均水平，力争调控在103.5%左右；地方财政收入增长15%；城乡居民收入实际增长7%；地区生产总值增长9%，人均地区生产总值超过8000美元。

　　(一)高质量、高水平做好奥运筹办和服务保障工作。

　　办好2008年奥运会、残奥会，是国家的大事，是全国人民的大事，是中华民族的大事，倍受世界瞩目。要坚决落实党的十七大和中央关于办好2008年奥运会、残奥会的指示精神，深入实践“绿色奥运、科技奥运、人文奥运”理念，大力弘扬在奥运筹办过程中形成的为国争光的爱国精神、艰苦奋斗的奉献精神、精益求精的敬业精神、勇攀高峰的创新精神和团结协作的团队精神，全面完成各项筹办任务，全力以赴做好各项服务保障工作。

　　精心打造一流的奥运工程。深入落实“五统一”要求，确保国家体育场按时竣工和测试赛成功举行，抓好国家会议中心、奥运村、媒体村、多功能演播塔及奥林匹克森林公园等相关设施的建设和交付使用。对全部场馆和设施进行反复测试，排查可能出现的故障点、风险点，逐项整改完善。督促业主单位与施工单位、设备供应商、服务提供商签订责任状，组建奥运期间的工程保障团队。完成首都国际机场扩建、北京南站改扩建和地铁10号线一期(含奥运支线)、机场线及奥运场馆周边道路建设任务。建成机场二通道等6条高速公路和安立路大容量快速公交线路。建成太阳宫、郑常庄燃气热电厂，完成奥运场馆及周边热力管线和输变电工程，确保奥运会能源供应的安全可靠。加快无障碍设施建设，满足举办残奥会的需要。

　　切实搞好城市环境建设。实施第十四阶段控制大气污染措施，执行最严格的污染物排放标准，加大夜间机动车污染专项整治力度，加强施工扬尘管理，取消城乡结合部地区的原煤散烧，完成油气回收治理改造、城区平房区5万户“煤改电”任务。认真落实《第29届奥运会北京空气质量保障措施》，密切与周边省市的环境合作，采取更加严格的控制手段，加强对环境质量的监控和预报，实现我们对国际社会的承诺。做好供水系统改造和城市水系骨干河道维护管理，改善城市水环境，确保用水安全。落实奥运城市环境景观规划，实施好2008添彩工程。继续抓好五大秩序专项治理，完成重点地区的环境综合整治工作。建立城市环境秩序监测和评价体系，完善长效管理机制，营造干净整洁、文明优美的城市环境。

　　高标准保障城市运行。有条不紊地推进奥运会筹备向举办转轨，在“2008环境建设指挥部”基础上组建城市运行指挥中心，建设城市运行指挥平台，建立高效的工作机制，实现各部门、各区县与奥组委工作的全面对接。积极配合奥组委安排好各项赛事，精心组织好奥运火炬传递和奥运会、残奥会开闭幕式等大型活动。全力做好奥运会、残奥会期间对外宣传、交通通信、住宿餐饮、水电气热供应、环境卫生、医疗卫生、志愿者队伍等工作。强化属地管理，健全场馆外围保障与场馆运行的协调机制，确保场馆运行和城市运行的无缝衔接。完善各类应急预案，加强实战演练，实施风险控制和动态管理，有效预防和处理各类突发事件。全面开展城市排水系统的排查和疏通改造，提高应对极端天气影响的快速反应能力。进一步完善奥运交通保障方案，科学组织、合理实施交通管理措施，加大公共交通服务供给，实现赛事交通与社会交通和谐运转。

　　确保首都安全稳定。全面落实奥运安保的总体方案和各项措施，建立高效畅通的指挥决策机制，加强安保合作和队伍建设，提高实战能力和服务水平。深入开展安全隐患排查整改，加强各类人员密集场所的安全检查，加大安全生产管理监督和源头治理力度，防范遏制重特大事故。做好信息安全工作。强化食品药品安全监管体系建设，健全食品药品可追溯系统，巩固产品质量和食品安全专项整治行动成果。健全以属地管理为主的维护稳定工作体制，逐级签订安全稳定责任书。强化社会治安综合治理，创新群防群治工作机制，有效预防、依法严厉打击各类违法犯罪活动，净化社会治安环境，确保实现“平安奥运”的目标。

　　努力营造热情友好的奥运氛围。深入开展“迎奥运、讲文明、树新风”活动，扎实推进人文奥运行动计划。实施文明礼仪示范工程，突出抓好公共卫生、公共秩序、公共交往等领域文明行为规范，着力提高窗口行业单位的业务技能和服务水平。强化赛场文明建设。尊重、关爱残疾人，在全社会形成扶残助残的良好风尚。加强首都公共文明示范地区建设，深入开展群众性精神文明创建活动。广泛动员全市人民行动起来，当好东道主，弘扬奥林匹克精神，展示优秀传统文化，展示市民文明素质，展示首都文明形象，共建共享奥运成果。

　　(二)保持首都经济又好又快发展。

　　做好2008年的经济工作，对于成功举办奥运会，保持首都经济又好又快发展，着力改善民生、促进社会和谐稳定，具有特殊重要的意义。要认真贯彻落实中央宏观调控的决策、部署和措施，坚持好字优先、稳中求进，立足于实现全面协调可持续发展，加快经济发展方式转变，进一步增强发展的协调性，不断提高首都经济的综合实力。

　　合理调控固定资产规模和结构。初步测算，2008年全社会固定资产投资总规模将达到4300亿元，增长11%左右。市政府固定资产投资拟安排264.5亿元，增幅与2007年持平。按照区别对待、有保有压的要求，发挥政府投资和规划的引导作用，在保证举办奥运等重大投资需求的基础上，继续加大对重点基础设施、重点产业以及关系民生的重点领域的投入，引导资金投向生态涵养发展区、城市南部、西部地区，支持新农村和以通州、顺义、亦庄为重点的新城建设。严格执行能耗和环保标准，加强和规范新开工项目管理。从严控制新增建设用地，强化监督检查，严肃查处违法违规用地。按照“建设一批、落地一批、储备一批”的思路，认真做好统筹协调和服务工作，切实抓好重点工程建设。

　　努力扩大消费需求。初步测算，2008年社会消费品零售额将接近4300亿元，增长13%左右。继续提高城乡居民收入水平，着力解决农民、企业职工及退休人员、城镇低收入群体的增收和社会保障问题，落实好相关政策措施，稳定居民消费预期，提高居民消费能力。全面落实商业服务业迎奥运三年行动计划，促进重点综合商业街、特色街区改造。支持银行卡工程，提高消费便利程度。加快农村现代流通体系和商业网点建设，改善郊区休闲旅游环境，进一步扩大农村消费。积极引导住房、汽车合理消费，扩大旅游、文化体育、时尚休闲的消费份额。同时，高度重视、切实做好居民基本生活必需品的价格稳定工作。加强与主要供应地的沟通与合作，搞好产运销衔接和重要商品储备，确保基本生活必需品不脱销、不断档。健全价格监测、预警和应急机制，完善防止价格异常波动预案。严格控制政府定价和政府指导价的调整，必要时对部分重要的居民基本生活必需品及服务，实行临时价格干预措施。加强市场监管，整顿和规范市场经济秩序，严惩价格违法和扰乱市场行为。采取应急救助、临时补贴等措施，确保低收入群体生活水平不下降，保障学校食堂食品供应和饭菜价格稳定。采取分类指导的办法，防止奥运会期间相关商品和服务价格出现恶性上涨。加强对经济运行的监测和调节，完善煤电油气运突发事件应急体系，做好能源和重要原材料的供需衔接，保障首都经济平稳运行。

　　深入推进产业结构调整。坚持以现代服务业为引领，完善产业导向政策和扶持政策，大力发展生产性服务业和文化创意产业。完善金融业扶持政策和服务体系，加快金融后台服务园区建设，积极吸引各类金融机构在京发展。推进空港综合保税港区等重点项目，促进现代物流和商务服务业发展。构筑投融资服务平台，培育骨干企业和战略投资者，搞好文化创意产业集聚区建设，壮大文化创意产业集群。紧抓奥运机遇，积极促进体育产业、会展业、旅游业加快发展。以高技术产业带动现代制造业发展，搞好项目引进和自主研发，推进第三代移动通信、数字电视、下一代互联网等关键技术的产业化应用，加快康宁玻璃基板、中芯国际增资扩产、现代汽车第二工厂、康明斯发动机等重点项目建设。支持发展生物医药、清洁技术和高端建筑建材产业。研究制定促进高端产业功能区发展的意见，加快基础设施和配套服务设施建设，提升产业集中度、竞争力和辐射力。做好搬迁后首钢原址的发展规划。健全管理服务机制、沟通协调机制和资源整合机制，为中央在京企业发展提供便利、快捷的全程服务。认真完成好第二次经济普查的各项任务。

　　不断提高自主创新能力。认真总结中关村科技园区20年发展经验，推动体制、政策、管理和组织创新，积极开展投融资、信用、协会组织、政府采购等各项改革工作，鼓励创业投资发展，深化非上市公司代办股份转让试点，推进知识产权质押贷款和信用贷款工作，进一步发挥园区的龙头带动作用。完善落实促进自主创新的各项政策，重点支持具有自主品牌的企业和项目发展，加快推进以企业为主体、市场为导向的各类新型产学研合作，努力为中央在京科研院所、高等院校发展创造更好条件，支持中央企业在京设立研发机构，积极促进国家重大科技创新项目落户北京。强化创新资源整合，完善首都科技条件平台运行机制，推动产业技术联盟建设，加大对引进技术的消化、吸收与再创新。加强知识产权保护，建设全国技术交易中心，支持留学人员创业园发展，促进大学科技园和孵化器与风险投资结合，探索建立促进成果转化的“种子”资金，健全科技成果产业化服务体系。抓好创新型人才培养和引进，畅通人才绿色通道，建立海外学人中心，着力解决高端人才在京住房、配偶就业、子女入学等方面问题。实施中长期科技发展规划，继续深化科技体制改革，围绕解决首都发展的难题，组织实施一批重大科技项目，争取在重点领域和关键技术上取得突破，提升首都自主创新能力。

　　继续做好节能减排和循环经济发展工作。建立市、区县政府节能降耗减排工作责任制，落实国家节能减排统计监测及考核实施方案和办法。继续调整退出高污染、高能耗、高水耗企业，支持首钢完成年度压产任务，推进化工二厂、有机化工厂搬迁，关停二热、三热的小火电机组。加强石化、建材、电力等重点行业的资源消耗管理、污染预防和全过程控制，完善能耗、水耗指导指标和监测平台。严格执行新增建筑节能设计标准，加强大型公建的能耗控制，推进既有建筑及供热系统节能改造，继续实施郊区新城集中供热工程。抓好清洁能源和可再生能源重点项目建设。完善用水计划、定额管理和超定额累进加价制度，发展节水灌溉10万亩。加快污水和再生水管网建设，对现有污水处理设施进行扩容改造。加强回收利用体系建设，积极推行垃圾分类收集和处理，推进再生资源产业化。搞好循环经济示范项目，加强产业园区生态建设。各级政府要带头树立资源节约意识，落实节能减排任务；加强宣传和引导，形成全社会节能减排的良好风尚。

　　加强社会主义新农村建设。坚持和完善各项强农惠农政策，推进新农村建设模式创新。2008年市政府拟安排“三农”资金150.9亿元。加快都市型现代农业区域化、规模化发展，扶持一批农业产业化项目，建设一批农业主题公园，积极培育走廊经济、流域经济，形成产业集群。鼓励农民自主创业，发展壮大集体经济，积极支持小城镇建设。健全农业支持、保护与服务体系，研究制定《农民专业合作社法》实施细则，加强基层农业技术推广体系建设，扩大农业政策性保险的险种和覆盖范围，加强农产品质量安全监管，提高动植物疫病防控能力。完成300个村庄规划编制任务，启动200个村庄基础设施“五项工程”建设，鼓励各区县根据实际情况加快建设步伐。完成70万农民安全饮水工程，升级广播电视“村村通”系统，加强农村消防设施建设，改造农村配电网。继续实施“亮起来、暖起来、循环起来”工程。加强农村公共服务中心建设，推进农村信息化，建立农村公共服务设施管护长效机制。大力促进生态涵养区发展，研究制定产业发展规划，提高市政府固定资产投资比重，推动一批符合功能定位的重大项目落户，支持资源型产业调整地区发展新兴产业，做好小流域综合治理、废弃矿山生态修复和新一轮山区搬迁工程等工作，探索建立区县结对帮扶、互利合作的长效机制。做好第七届中国花卉博览会各项筹备工作。

　　深入推进经济体制改革。完善市和区县财政管理体制，健全财政转移支付制度，提高一般性转移支付的规模和比例，推进基本公共服务均等化，促进区县功能定位的落实。全面推行非税收入收缴管理改革。开展社会领域投融资体制改革试点，完善政府投资管理模式。深化国有企业改革，支持一级企业引进战略投资者，鼓励非公资本参与二、三级企业股份制改造，加大国有资本从劣势企业退出的力度，推进企业上市和上市公司重组。继续探索国有资本经营预算管理，开展国有独资公司完善法人治理结构的综合试点。深化集体企业改革。鼓励、支持和引导非公有制经济健康发展，安排专项资金5亿元，支持中小企业发展，完善面向中小企业的融资体系，研究设立相应的再担保机构。进一步加强社会信用体系建设。积极推进供热体制改革。巩固完善生态林补偿机制，探索水源保护区、矿产资源保护区生态补偿机制。搭建农地流转信息平台，抓好集体建设用地流转试点。大力推进农村综合改革，开展城乡结合部地区综合管理体制改革试点，支持放宽农村金融机构准入政策试点工作，研究完善整建制农转居的配套政策，深化乡村集体经济产权制度改革，积极推进集体林权制度改革。

　　着力提高对外开放的质量和水平。建立利用外资区域与产业评估体系和质量评价指标体系。继续做好重点地区的投资促进工作，大力引导外资投向生产性服务业、文化创意产业和高端制造业，吸引国内外大企业在京设立地区总部等机构。制定促进服务贸易发展的政策措施，抓好各类服务外包基地建设，积极承接国际服务外包的高端环节。鼓励进出口交易活动，继续支持拥有自主品牌、自主知识产权的产品出口，拓展文化产品和文化服务的对外贸易，扩大先进技术设备和资源性产品进口。支持有条件的企业开展跨国经营，培育国际名牌和国际企业。认真做好第七届亚欧首脑会议等国家重大活动的外事服务工作。积极参与京津冀、环渤海区域经济分工合作，促进区域协调发展。

　　(三)加快推进以改善民生为重点的社会建设。

　　着力解决民生问题，着力化解社会矛盾，着力加强社会管理，着力营造欢乐祥和的社会环境，对促进社会和谐、办好奥运会具有重要作用。要坚持以人为本，推进基本公共服务均等化，为群众办好59件重要实事，努力实现“无零就业家庭、无城镇危房户、无重大重复上访户、无社会救助盲点、无拖欠工资问题”的目标，更好地改善人民生活。

　　优先发展教育。加大投入力度，支持100所小学在主要项目上达到新颁办学标准，进一步改善农村学校、薄弱学校办学条件，发展农村幼儿教育，推进山区义务教育提升工程。完善农村地区学生、城市困难家庭学生、特殊教育学生的助学政策，保证来京务工人员子女接受义务教育。继续组织城镇教师和大学生到农村支教。全面加强教师队伍建设，设立40个农村中小学教师研修工作站，保证1000名农村骨干教师进站研修。提高农村特别是山区教师工资待遇水平，进一步改善山区教师工作学习条件。大力实施素质教育，稳步推进课程改革，减轻中小学生课业负担，提高教育质量。加强学校体育工作和心理健康教育，提高学生综合素质。加快职业教育基地和示范性院校建设，促进中高职教育相衔接。实施高等学校“质量工程”与“创新工程”，加快沙河、良乡高校园区建设。加强在职培训、社区辅导等继续教育。鼓励、引导和规范民办教育事业健康发展。推进学生德育实践活动基地建设，加强和改进大学生思想政治工作，完善学校、家庭、社会相结合的未成年人教育网络。

　　进一步扩大和促进就业。认真落实《就业促进法》，健全小额贷款、创业培训等扶持政策，大力支持自主创业和自谋职业。完善“零就业家庭”就业动态帮扶机制，做到出现一户、帮扶一户、解决一户，保证有就业能力的家庭至少有一人就业，实现“无零就业家庭”目标。做好首钢等国有企业富余职工分流安置工作，引导和鼓励高校毕业生到基层和农村就业，扩大残疾人就业规模。实行农村劳动力转移就业登记动态管理制度，将促进就业政策进一步向农村延伸，实现7万名农村劳动力转移就业。以健全村级就业服务体系为重点，创建2000个充分就业社区(村)。加强高技能人才培养，重点做好面向农民、就业困难群体的职业技能培训。落实《劳动合同法》和《劳动争议调解仲裁法》，健全劳动关系协调机制，保障劳动者合法权益。完善企业工资支付监控、工资支付信用和工资保证金制度，坚决制止企业拖欠工资行为，努力做到“无拖欠工资问题”。

　　加快建立覆盖城乡的社会保障体系。2008年市级财政拟安排社会保障支出62.1亿元，比上年增加8.6亿元。继续完善城镇职工基本医疗保险制度，调整职工基本医疗保险报付结构，提高门诊特别是社区用药报销比重。完善“一老一小”医保政策，解决部分门诊药费报销问题，增加大病医保种类。加大政府补贴力度，完善新型农村合作医疗统筹模式和补偿政策。出台劳动年龄内无医疗保障城镇居民参加医疗保险的政策，实现城乡医疗保障制度的全覆盖。推行医保报销“一卡通”。逐步做实基本养老保险个人账户，提高企业退休人员基本养老金水平。实施新型农村社会养老保险改革方案，落实城乡无社会保障老年居民养老保障办法，为符合条件的70万老年人每人每月发放200元福利养老金，基本实现城乡养老保障制度的全覆盖。认真研究低保边缘群体的保障问题，完善社会救助制度，健全对低收入人群的救助措施，实现社会救助全覆盖，做到“无社会救助盲点”。加强养老和福利设施建设，提高居家养老服务水平。大力发展慈善事业。

　　多渠道解决群众住房困难。加快健全廉租住房制度，2008年市级财政筹集资金29亿元，建设收购50万平方米廉租住房，将廉租住房保障范围扩大到低收入住房困难家庭。加强经济适用住房的建设和管理，建设300万平方米经济适用住房。增加中低价位、中小户型普通商品住房供应，重点建设450万平方米“两限”房。完善建设的协调推进机制，加快审批进度，确保项目落地、土地供应、资金投入、税费减免和工作责任落实到位，在保证质量的前提下，尽快形成实物供应。统筹旧城区风貌保护和解决群众住房困难，修缮整治40条胡同、1400个院落；安排市级财政支出5.8亿元，全力以赴解决1万户居民危房问题，落实排险修缮责任，改善居住在五类危房中群众的住房条件，努力实现“无城镇危房户”的目标。完成300个老旧小区的改造任务。进一步加强和改进房地产市场调控，调整土地供应结构，严格执行住房信贷管理政策，加快“二手房”和住房租赁市场发展，抑制房价过快上涨。

　　推进卫生事业改革与发展。研究落实医药卫生体制改革的具体方案和措施。完善公共卫生服务体系，增强对重大疾病的防控能力，深入推进爱国卫生运动，倡导健康生活方式，提高市民健康水平。强化社区卫生服务功能，推行社区卫生首诊制、双向转诊制和家庭医生服务模式，推广中医药服务。完善医疗服务体系，加快郊区医疗中心建设，完成所有社区卫生服务中心(站)的标准化建设和基本设备配备任务，加强社区医护人员队伍建设，推进优质医疗资源向郊区转移。改善医院服务与管理，使各项缓解群众看病就医困难的措施落到实处。完善药品供应保障体系，拟安排市级财政支出2亿元，扩大社区基本用药目录，增加品种，实现零差率药品配送覆盖城乡。解决好乡村医生基本待遇和养老保障问题。重视妇幼卫生和计划生育事业，保持稳定的低生育水平。研究制定老年医疗服务体系、精神卫生防治体系建设规划。

　　继续推动公交优先发展。加大公共财政的投入力度，在加快轨道交通建设的同时，推进地面公交提级改造。重点建设交通枢纽和综合驻车设施，改善换乘条件，提高运输效率。推进城乡客运一体化进程，改造乡村公路200公里，开通农村客运线路25条，实现行政村全部通公交。按照整体推进、分级负责、分步实施原则，将公交低票价政策逐步向郊区农村延伸。组织实施缓解市区交通拥堵第五阶段措施，建设好交通综合信息平台和智能交通管理系统，提高管理水平，为市民提供更快捷、方便的出行服务。

　　大力发展文化事业。大力弘扬社会主义核心价值，加强和谐文化建设，培育文明风尚。制定新的基层文化建设规划，建设一批街道、乡镇综合文化站和行政村文化活动室。办好“文艺演出星火工程”、“周末场演出计划”、“民族艺术进校园”等活动，精心举办系列奥运文化广场演出，培养基层文化活动带头人。继续推动益民书屋建设。加快国家话剧院、首都图书馆二期暨北京市方志馆工程、市文化中心等重要文化设施建设。加快经营性文化事业单位转企改制，培育一批文化精品，繁荣文化市场。保护民族民间优秀文化，开展文物与非物质文化遗产普查。整合首都文化资源，发挥整体优势，大力发展广播影视、新闻出版事业，繁荣哲学社会科学。加强科普工作，重点普及节能、节水、环保知识，提高公众科学素养。加强网络文化建设和管理，营造良好网络环境。广泛开展全民健身运动，所有行政村配建全民健身设施。进一步提高竞技体育综合实力，力争在奥运会、残奥会上取得优异成绩。

　　全面提高社会管理水平。研究制定首都社会建设总体规划，健全组织领导体系，鼓励和支持各类社会组织的发展，建立以社区为基础、社会组织为载体、社会人才队伍为保障的社会公共治理体制。着力加强基层基础工作和社区建设，探索新的社区管理体制，建立社会工作者队伍，健全社区工作者职业资格、工资增长及培育管理机制，规范社区公益事业经费使用和管理，改善社区办公和服务用房条件。完善政府购买社区公共服务政策，创新政府公共服务的提供方式。建设好信息系统，健全房屋租赁和居住证管理体系，提升流动人口服务管理水平。完善信访制度，加强基层信访工作，充分发挥社会矛盾调处中心、信访信息系统、非紧急救助服务系统等平台的作用，畅通群众利益诉求表达渠道。健全人民调解、行政调解、司法调解的衔接机制，加强行业调解、社区调解组织建设，发挥律师等社会资源的作用，提高综合化解社会矛盾的效率和效果。主动帮助群众排忧解难，依法妥善处理人民内部矛盾，重点解决重大重复上访积案，努力把各类矛盾化解在萌芽状态、化解在基层，实现“无重大重复上访户”目标。

　　全力维护团结和谐的大好局面。深入开展民族团结进步创建活动，加快少数民族乡村经济发展。支持爱国宗教团体自身建设，提高依法管理宗教事务的水平。进一步调动归侨侨眷和海外侨胞参与北京建设的积极性。加强对台工作，推进京台经济科技文化交流与合作。依法维护妇女儿童、残疾人的合法权益。认真落实“五五”普法规划，积极推进公益法律服务体系建设。支持工会、共青团和妇联等群众组织开展工作。充分发挥国防教育基地和大众传媒作用，深入开展丰富多彩的双拥共建活动，加强国防后备力量建设，继续做好优抚安置工作，努力解决随军家属就业和子女入学等问题，不断巩固军政军民团结。

　　(四)加强政府自身建设。

　　深化行政管理体制改革，加强政府自身建设，是贯彻落实科学发展观的必然要求，是履行好政府职能、优化发展环境的关键环节，是推动科学发展、促进社会和谐的重要保障。必须进一步加强政府自身建设，努力建设服务型政府。

　　认真学习贯彻党的十七大精神。我们要紧密结合北京工作实际，学习好、领会好、贯彻好、落实好党的十七大精神，积极投入到学习实践科学发展观的活动中去，不断提高领导科学发展的能力。要努力把握首都经济社会发展的客观规律，努力掌握统筹兼顾的根本方法，努力提高决策的科学化和民主化水平，以改革创新精神开拓各项工作的新局面。

　　不断提高依法行政能力。全面执行人民代表大会及其常委会的决议，自觉接受人大监督。认真听取民主党派、工商联、无党派人士和各人民团体的意见，接受人民政协的民主监督。接受新闻舆论和社会公众监督。深入贯彻国务院全面推进依法行政实施纲要，加强行政监督，规范行政行为，完善和严格执行政府绩效管理制度和行政问责制度，加大对滥用权力和失职渎职行为的追究力度。完成年度行政立法任务，理顺行政执法体制，提高行政执法水平。

　　着力转变政府职能。积极推进行政管理体制改革，强化社会管理和公共服务职能，精简和规范各类议事协调机构及其办事机构，制定事业单位分类改革总体方案。深化行政审批改革，继续减少和规范行政审批和许可事项，推进政府管理机制、制度和服务方式创新，更好地为基层、企业和社会公众服务。落实国务院《政府信息公开条例》，健全政府信息公开工作机制和制度规范，增强政府工作透明度和公信力。

　　大力加强作风建设。深入开展调查研究，了解工作中的突出问题和群众最关心的利益问题，总结推广实践中创造的好经验好办法，使我们的各项工作更加符合客观实际，更加体现群众意愿。加强部门间的沟通协调配合，着力提高行政效率，力戒推诿扯皮。坚持求真务实，切实精简会议，减少文件，狠抓工作落实。坚持勤俭办一切事情，降低行政成本，反对铺张浪费。加强公务员队伍建设，增强公仆意识，全心全意为人民服务。

　　切实推进廉政建设。加强对权力运行，特别是对领导干部、人财物管理使用、关键岗位的监督和制约。规范财政转移支付、土地征用出让、政府采购、国有资产转让等公共资源权力运行。深入开展执法监察、廉政监察和效能监察，加强制度建设，积极探索从源头上预防和治理腐败的新举措。坚决纠正损害人民群众利益的不正之风，坚决查处违法违纪案件，决不姑息。

　　各位代表，举办一届有特色、高水平的奥运会、残奥会，把北京建设成为繁荣、文明、和谐、宜居的首善之区，是中央的重托、人民的期待、民族的愿望，是我们无比神圣的使命和义不容辞的责任。让我们紧密团结在以胡锦涛同志为总书记的党中央周围，高举中国特色社会主义伟大旗帜，以邓小平理论和“三个代表”重要思想为指导，深入贯彻落实科学发展观，在中共北京市委的领导下，大力发扬改革创新、求真务实、团结奋进的精神，扎实工作，奋力拼搏，把首都改革开放和现代化建设不断推向前进！
